# RecoveryWorks: vocational support services for recovery capital growth

**DOI:** 10.3389/fpsyt.2026.1751052

**Published:** 2026-05-01

**Authors:** Toby Lynch, David Best, David Eddie, Vinod Rao, Joseph LaFratta, Estee Sharon

**Affiliations:** 1 Department of Psychiatry, Massachusetts General Hospital, Boston, MA, United States; 2 Harvard Medical School, Boston, MA, United States; 3 Research, Stronger Together Around Recovery (STAR) Recovery, Recovery Outcomes Institute, Hope Valley, United Kingdom; 4 Recovery Research Institute, Boston, MA, United States

**Keywords:** addiction, employment, psychology of working, recovery, recovery capital, vocational rehabilitation, substance use disorders, career counseling

## Abstract

This paper introduces RecoveryWorks, a multi-system recovery and vocational support program designed to expand the therapeutic landscape of recovery by increasing access to career development resources that support meaningful employment. In contrast to vocational approaches that prioritize rapid job placement, RecoveryWorks embeds work within a recovery-oriented system of care. By linking the underutilized human capital of individuals in recovery to community resources, the program aims to generate reciprocal gains in personal and community recovery capital over time.

## Introduction

Contemporary models of addiction recovery emphasize both symptom remission and community reintegration, with employment recognized as a central pillar (1). Despite persistently low employment rates and high interest in working among individuals in addiction treatment vocational supports remain difficult to access (2–4). When available, services often prioritize rapid job placement over career development (5). Accordingly, scholars have called for a broader focus on career development and meaningful employment—work perceived as personally or socially significant—to support sustained recovery (6, 7).

This paper introduces RecoveryWorks, a multisystem recovery and vocational support program designed to expand the therapeutic landscape of recovery (8) by increasing access to career development resources that support meaningful employment and, in turn, survival and sustained recovery (9). In contrast to vocational approaches that prioritize rapid job placement, RecoveryWorks embeds work within a recovery-oriented system of care. By linking the underutilized human capital of individuals in recovery to community resources, the program aims to generate reciprocal gains in personal and community recovery capital over time (1, 10).

## Theoretical frameworks

RecoveryWorks integrates Psychology of Working Theory (PWT; 9) with recovery-oriented models of Recovery Capital (11) and C-CHIME (Community–Connection, Hope, Identity, Meaning, and Empowerment (12)).

## Psychology of working theory

Within PWT, career adaptability refers to the capacity to self-regulate in response to career transitions and comprises career concern (future-oriented planning), curiosity (exploration of vocational possibilities), control (autonomous career decision-making), and confidence (self-efficacy in overcoming barriers and achieving goals (13)). Career adaptability is shaped in part by work volition, defined as perceived freedom to make career choices (9). Together, work volition and career adaptability influence access to decent work that meets basic psychological needs for autonomy, competence, and relatedness as outlined in self-determination theory (14).

## Recovery capital

Recovery capital refers to resources that can be mobilized to initiate and sustain recovery and is commonly conceptualized across personal (e.g., coping skills, self-efficacy), social (e.g., recovery-supportive networks), and community domains (e.g., access to resources and non-stigmatizing community attitudes (15)). Negative recovery capital refers to constraints on recovery operating in community (e.g., stigma), social (e.g., discrimination, exclusion), and personal domains (e.g., internalized stigma (15)).

## Community–connection, hope, identity, meaning, and empowerment

C-CHIME, an adaptation of the CHIME model of mental health recovery, describes how recovery capital is transmitted through communities through social connection and subjectively experienced through personal recovery processes of hope, identity, meaning, and empowerment (12).

## The RecoveryWorks program

Although employment has been shown to support social connectedness and activate the personal recovery processes described in C-CHIME (16–21), many individuals in recovery, particularly those in treatment or early recovery, experience constrained work volition and view work primarily as a means of survival (22, 23). To increase access to meaningful employment, RecoveryWorks provides bridging social capital as a scaffold for developing career adaptability resources and initiating personal recovery processes. In samples of individuals receiving addiction treatment and vocational support, career adaptability has been associated with hope and with access to decent work that supports psychological need satisfaction. In turn, need-satisfying work has been linked to work-related identity commitment, meaning construction, and psychological empowerment (24–27).

RecoveryWorks extends beyond the individual by aiming for a mutually reinforcing cycle of growth in personal and community recovery capital. Meaningful employment creates opportunities for contact between workers with stigmatized identities, including individuals in recovery, and those without such identities, thereby increasing workplace cohesion and reducing stigma within both workplaces and the broader community (28–32). Employees in recovery have been reported to miss fewer workdays than individuals with untreated substance use disorders and to show slightly lower absenteeism than the general workforce (33). Employment among individuals in recovery also generates economic benefits, including greater access to employer-sponsored health insurance, increased capacity to support families through earned income, and tax contributions (33–35). Although direct evidence remains limited, these benefits may be greater when employment reflects strong person-environment fit and supports basic psychological needs. Over time, it is expected that community gains reflected in increased cohesion and empowerment will feed back to strengthen personal recovery capital through increased work volition, supporting career adaptability, and reactivation of personal recovery processes.

## RecoveryWorks components

RecoveryWorks is structured around five interdependent service components: mentorship, career coaching, self-regulatory skills training, peer support, and Recovery Celebration Events. Together, these components are designed to enhance work volition and career adaptability and to activate personal recovery processes during return-to-work transitions.

Mentorship and career coaching are available for the duration of enrollment (up to five years), consistent with longitudinal evidence suggesting that recovery processes often become more autonomously sustained after this period (36, 37). Access to peer support and invitations to Recovery Celebration Events are extended indefinitely. Except for Recovery Celebration Events, all services are delivered outside clinical addiction treatment systems via a Zoom-based virtual platform. Communication and care coordination among service providers are supported through monthly team meetings via Zoom and access to a secure file via Microsoft teams.

### Mentorship

Mentorship is provided through weekly 30-minute sessions throughout enrollment (38). Drawing on life design and narrative counseling principles, which have been linked to career adaptability (39–41), mentorship emphasizes career-related activity followed by co-reflection on internal and external feedback. Career-related activity, reinforced by the mentor–mentee working alliance, supports identity change through social learning, as mentors model professional behavior and reinforce an emergent prosocial “worker” identity. Co-reflection supports meaning by helping participants deconstruct stigmatizing narratives and reconstruct them to include more adaptive, recovery-oriented themes.

Mentors also provide assertive linkage to resources within and beyond the RecoveryWorks community network. Within RecoveryWorks, mentors connect participants to individual career coaching—accompanying them to initial sessions as needed to support engagement and continuity—and to the self-regulatory skills training group via email introductions and checking and reinforcing attendance. Assertive linkage beyond the RecoveryWorks community network is grounded in Asset-Based Community Engagement and involves systematically identifying, mapping, and mobilizing community resources (42) (e.g., mutual aid groups, housing supports, legal assistance, collegiate recovery programs (43), and recovery-friendly employers (44).

Mentors are expected to maintain a working knowledge of community resources through prior program participants and ongoing community engagement and to share resource information with one another during weekly meetings (45, 46). Mentors hold at least a bachelor’s degree, have a minimum of five years of experience in recovery-related services, and demonstrate knowledge of recovery principles and pathways. They may be recruited from the program’s participant pool and draw on lived experience to transmit hope, consistent with peer recovery traditions of offering “experience, strength, and hope” (47).

### Career coaching

Career coaching is initiated at participants’ request, in coordination with their mentor, following clarification of career goals. Coaching targets anticipatory vocational socialization through exposure to normative workplace expectations (48), training in job-search competencies (e.g., résumé development and job interviewing (49)), and job search self-regulation (50). Career coaches hold at least a bachelor’s degree and generally have an average of five years of coaching experience, with an expressed interest in working with individuals in recovery. A potential benefit of coaches’ relative lack of prior experience working with individuals in recovery is that it may blur boundaries between stigmatized and non-stigmatized groups and create opportunities for participants’ work-related identities to be affirmed by nonclinical others with whom they do not typically interact in institutional contexts (51, 52).

### Self-regulatory skills training

The Group for Recovery-Oriented Work (GROW) is a 16-session group-based intervention grounded in mindfulness (53–55), narrative therapy (56), positive psychology (57), and job crafting (58). The job crafting intervention was designed to facilitate self-determined modification of cognitions, and behaviors to improve person-environment fit and resolve discrepancies between “real” (actual) and “ideal” (desired) levels of psychological need satisfaction (59). GROW skills are organized as promotion-oriented (aimed at accumulating recovery capital) or prevention-oriented (aimed at buffering negative recovery capital (60, 61)). Participants are encouraged to adopt a dialectical stance, consistent with mindfulness practice, to maintain regulatory focus flexibility—that is, the capacity to shift between promotion and prevention in response to changing contextual demands. GROW is co-facilitated by a master’s-level clinician who is trained and certified in the GROW model and employed in addiction treatment, together with a peer in recovery.

### Peer support

GROW sessions provide structured opportunities for peer support, which has been associated with hope (62) through social learning and social control mechanisms (63). After completing GROW, participants may continue peer support through a monthly group and a closed online forum for resource sharing and mutual support.

### Recovery celebration events

Consistent with conceptualizations of recovery as a social contagion and aligned with initiatives such as the Inclusive Recovery Cities model, quarterly Recovery Celebration Events convene RecoveryWorks participants and service providers, family and friends, addiction treatment providers, advocacy organizations, community leaders, employers, and other stakeholders (1, 64, 65). These events are designed to facilitate connection across diverse community sectors, increase the visibility and attractiveness of recovery, elicit referrals, affirm recovery as valued human capital, and strengthen pathways for assertive linkage. As the program’s sole in-person component, Recovery Celebration Events are integral for informal networking and community support.

### Structured assessment

Participant progress is monitored through quarterly structured assessments. Surveys collect data on recovery- and career-related changes. Measures include the R1 Recovery Capital Screener-36 (RCs-36)* instrument, a widely used strengths-based tool in addiction recovery settings, and the Career Adapt-Abilities Scale (13), which assesses career concern, control, curiosity, and confidence and has been validated in prior studies (66, 67). Monitoring is supplemented by session-level documentation: mentors and career coaches record attendance and engagement, salient recovery and career events, and behavioral observations after each contact. Survey data and progress notes are stored in a shared secure online file accessible to all service providers for ongoing case review.

## Return-to-work processes in RecoveryWorks

Participants are referred through community-based addiction treatment programs and recovery support organizations (e.g., peer recovery centers, departments of correction, recovery courts). Upon enrollment, participants receive a welcome message from the program director introducing their assigned support team. They then complete an initial virtual onboarding visit with their assigned mentor, which includes a program overview; clarification of staff roles, scopes of responsibility, and referral pathways; review of ethics, boundaries, and confidentiality; and an introduction to the recovery capital model.

Consistent with the Choose-Get-Keep model of vocational rehabilitation (68), RecoveryWorks conceptualizes return to work as an iterative, cyclical process. “Choosing” activities include career exploration, planning, and decision-making. “Getting” activities focus on job search and on building the skills and supports needed to obtain employment. “Keeping” activities focus on developing, sustaining, and deriving meaning from employment over time, while also supporting longer-term career development.

### Choosing

Career concern is elicited through co-reflection on assessment findings. Identified needs in career adaptability and recovery capital then guide subsequent career exploration, planning, and decision-making.

For example, Participant 1’s CAAS and REC-CAP scores indicated high, evenly distributed career adaptability (CAAS = 118) and closely aligned personal and social recovery capital, suggesting adequate social support. In contrast, the community recovery capital score suggested limited engagement with broad community resources. In mentorship, co-reflection on this discrepancy informed a career plan to explore paid temporary or provisional work through “splinting”—adopting a work-related identity to meet immediate survival needs—while simultaneously pursuing job-search activities oriented toward a closer person–environment fit (69).

GROW exercises (e.g., best possible future work self (70, 71)), skills training (e.g., clarification of values, interests, and strengths), and peer support that provides vicarious “possible work selves” are designed to translate career concern into curiosity and promote career exploration. These experiences may also enhance work volition by supporting shifts from stigma-based self-concepts (e.g., “addict,” “unemployed”) toward culturally recognized and personally meaningful potential future identities (e.g., “worker,” “professional” (72, 73)). Coaching then offers opportunities to “try on” these possible selves on a provisional basis (e.g., mock interviews) and to appraise internal and external feedback relevant to person–environment fit. Fit appraisal is further strengthened by GROW-based training in affect labeling, which helps participants use internal cues (e.g., emotional responses) and external feedback (e.g., social validation or rejection) to interpret affective states and guide career decisions (74, 75).

Career control is enhanced through co-reflection that helps participants refine provisional work-related identities and make decisions regarding career goals. Mentors provide information about accessible work identities that are compatible with, or complementary to, existing prosocial identities and offer psychoeducation on workplace conditions that tend to support recovery processes (e.g., autonomy-supportive supervision, values congruence, and an organizational view of employees as valued human capital (76–78)). Consistent with hope theory as articulated by Snyder (79), the pursuit of meaningful employment requires both a clear mental representation of a future work self and perceived pathways for overcoming barriers.

### Getting

Career control is also central to getting hired. While co-reflection on job-search activities helps identify discrepancies between current behavior and an imagined “best possible future work self,” which can be used to refine goals and clarify pathways, career confidence is enhanced through co-reflection on accumulating evidence of progress in resolving these discrepancies and training in job-search competencies through coaching (e.g., résumé development, interviewing, and personal branding (80–82)).

### Keeping

Autonomy-supportive supervision creates opportunities for job crafting, which, when successful, can strengthen psychological empowerment. Identity commitment is supported through behavioral crafting that expands and diversifies workplace group memberships (e.g., work teams, professional associations), thereby increasing resilience (83–85). Meaning construction is supported through mentor-guided co-reflection that deconstructs stigma-shaped narratives and, following crafting efforts, evaluates person–environment fit and organizes change around recovery- and career-relevant themes. As work-related identities become more culturally recognized and personally valued, such as “worker” or “professional,” the meaning attached to work may become increasingly redemptive. Evidence of gains in career control and confidence identified through structured assessment justifies the gradual, incremental transfer of responsibility for crafting activities from mentors to participants. As this autonomy-supportive scaffolding is reduced, participants are better positioned to sustain self-determined accumulation of recovery capital after program completion.

## Negative recovery capital

Stigma—defined by Goffman (86) as a socially discrediting attribute, behavior, or reputation—can be conceptualized as negative recovery capital that operates across personal, social, and community domains (87). In the community domain, negative recovery capital includes structural barriers and workplace hazards; in the social domain, it includes exclusion and stereotype threat; and in the personal domain, it includes internalized stigma.

## Negative community capital

### Workplace hazards

Individuals in recovery with low work volition may experience motivation for job search as primarily introjected or externally regulated. Pressure to meet survival needs or escape aversive internal states associated with controlled motivation may narrow career exploration and increase the likelihood of accepting any available job. In turn, poor person-environment fit may elevate risk of return to addictive behavior (88–90).

Hazardous workplaces are characterized by low levels of social control, such as limited supervision, high mobility, permissive substance-use norms, or ready access to substances due to high prevalence of employees who use them (e.g., some construction, transportation, and food service settings (91)) or conditioned cues linked to past addictive behavior. Alternatively, they may have job demands that are non-negotiable and exceed available coping resources so that work remains primarily controlled rather than self-determined. Non-negotiable job demands are common among individuals in early recovery and in addiction treatment. These demands may include poor working conditions, low wages, inadequate benefits, jobs perceived as lower in status than prior employment, long hours, inflexible schedules, multiple jobholding, and work-family conflict. Such demands are often not offset by workplace resources and may either limit access to coping resources outside of work or overwhelm them (19, 20). As a result, competing employment and recovery demands may generate role conflict and undermine stability across both work and recovery identity domains (92).

When demands exceed resources and work lacks intrinsic meaning, job strain and burnout increase, which may elevate risk of returning to use as a form of self-medication (93–95). As coping resources are depleted, employees in recovery are vulnerable to job insecurity, which threatens emergent work-related social identities and valued role identities linked to employment (e.g., provider (96)). The perception of not belonging within the workplace reduces help-seeking following returns to use or recurrence of psychiatric symptoms (97). Concealing recovery status, treatment involvement, or other stigmatized yet prosocial identities is often justifiable considering discriminatory or unfair treatment (98, 99). While it may reduce immediate exposure to these threats, it can also undermine needs for belonging over time (100, 101). Similarly, opting out of workplace rituals involving substances may protect recovery but can reduce career-relevant social capital (102).

Person-environment fit – particularly emotions-demand fit (103) – may require searching for employment in recovery supportive workplaces, either as a stepping stone or on a longer-term basis. Accordingly, mentors maintain relationships with, and working knowledge of, recovery-ready or recovery-friendly workplaces to support assertive linkage. First launched in New Hampshire and now implemented at state and local levels, the Recovery Friendly Workplace Initiative guides employers in adopting protections, policies, and practices that expand opportunities for individuals in recovery and support the integration of recovery within the workplace (44, 104). Mentors and participants may also explore values congruence in workplaces in which shared identity promotes informal social control, mutual accountability, and support. This includes workplaces that primarily employ individuals in recovery, such as therapeutic workplaces, which have been associated with greater hope and subsequent work engagement (62). It may also include unionized workplaces, where shared worker identity and member assistance programs can provide support and accountability even in the absence of explicit recovery peer support (105).

Psychoeducation in GROW includes information about workplace conditions associated with reduced strain, greater basic psychological need satisfaction, and accumulation of recovery capital. These conditions include fair treatment, recognition, skill variety, opportunities to develop and apply skills, fair compensation and advancement opportunities, workplace friendships, social support from coworkers and supervisors, and formal policies that deter substance use (106–111). GROW also provides psychoeducation about protections under the Americans with Disabilities Act (ADA), including the right to reasonable accommodations that support treatment engagement and recovery activities, such as scheduling flexibility to attend therapy, mutual-aid meetings, or court or probation obligations, unless doing so would impose an undue hardship on the employer (112).

### Structural barriers

Work volition is often diminished by structural career barriers. Mandated obligations (e.g., methadone dosing, court appearances, counseling sessions, parole check-ins) frequently conflict with standard work schedules, creating logistical strain (113–116). Immediate security needs—including limited transportation, caregiving responsibilities, and housing instability—may further constrain work volition and impair career decision-making (19–21, 117, 118). In addition, employment may jeopardize eligibility for public benefits or subsidized housing, which can contribute to ambivalence about career activities (114).

Within organizations, constraints such as criminal records, professional sanctions, and limited educational attainment restrict access to employment and advancement. Hiring practices—formal or informal—narrow opportunities by disqualifying applicants with résumé gaps or prolonged unemployment (114). These barriers disproportionately affect Black and Hispanic individuals, women, older adults, and individuals with co-occurring psychiatric conditions or substance-related convictions (18, 114). In addition, stigma and stereotyping in hiring and workplace contexts limit recruitment and advancement when employers perceive applicants in recovery as unreliable or unmotivated (22, 119–123).

RecoveryWorks addresses these barriers through both indirect and direct means. Indirectly, Recovery Celebration Events aim to counter stigmatizing narratives about recovery and work by increasing the visibility of employed individuals in recovery and their accomplishments. Attendees are encouraged to disseminate recovery-supportive norms and affirming messages within and across their social networks (1). Directly, RecoveryWorks advocates on behalf of participants who choose to disclose recovery status to prospective employers by providing references that attest to their employability.

### Unmet needs

Most addiction treatment programs remain oriented toward an acute-care model and do not offer vocational support services (124). Clinical staff often lack adequate training, and even when trained employment specialists are co-located within treatment, service delivery may be constrained by competing demands between clinical stabilization and vocational needs (125–127). Although existing services have been shown to facilitate job placement, concerns remain regarding job tenure and durability (128–130). RecoveryWorks addresses these unmet needs by providing vocational support to individuals in addiction treatment over a sufficient duration for work to begin functioning as a source of meaning. Additionally, virtual delivery expands access to individuals in treatment programs that lack such services and reduces common barriers to participation, including geographic isolation, transportation limitations, and scheduling constraints.

## Negative social capital

Social exclusion restricts access to career-relevant bridging social capital, limiting exposure to information and opportunities that would otherwise support career concern and curiosity (131). In turn, constrained access to prosocial networks and attachment insecurity can reinforce engagement with substance-using networks that meet needs but also reinforce negative cultural capital (132, 133). In addition to providing access to career-relevant bridging social capital through its vocational supports and assertive linkage to broader community resources, coaching and GROW mitigate the effects of social exclusion on workplace functioning by strengthening social-cognitive capacities that can be compromised among individuals with substance use histories. Training targets theory of mind and social problem-solving, as well as self-differentiation—balancing authenticity with belonging across social contexts (134–137).

Coercive systems, such as mandated treatment and criminal justice supervision can frustrate autonomy and foster controlled motivation, thereby undermining career control and confidence. In contrast, mentorship, specifically the mentor-mentee working alliance, and peer support offer protective social control that buffers risk of returning to addictive behavior under strain while gradually supporting autonomy development. To strengthen self-efficacy (138) and career control, GROW skills training targets career decision-making, such as decisions about disclosure of stigmatized identities, and applied problem-solving, including navigation of disability and employment law and access to workplace accommodations.

## Negative personal capital

Internalized stigma and anticipated discrimination can lead individuals in recovery to avoid thinking about the future, thereby constraining career concern (139). Experiential avoidance is intensified by hypervigilance to stereotype threat and by histories of career trauma, including involuntary job loss, loss of professional licensure, educational disruption, and prolonged unemployment (140–146).

To address this barrier, mindful reappraisal training in GROW is designed to strengthen regulatory focus flexibility, enabling participants to shift intentionally between promotion and prevention focus in response to changing contextual demands and career goals. Participants learn to identify automatic threat-based appraisals and use mindfulness skills, such as attention to breathing, to enter a broader metacognitive stance in which emotional reactivity is reduced. From this stance, they are better able to construct meaning from career activities that are not intrinsically motivating by linking them to broader recovery goals and to reframe perceived threats as challenges (147–149). GROW also includes mindfulness-based stress reduction training to increase resilience to job strain (150) and mindfulness-based relapse prevention training to reduce risk of return to addictive behavior under strain (151).

## Conclusion

Preliminary data collection, participant feedback, and field observations suggest that RecoveryWorks is feasible and acceptable; however, the formal empirical study has not yet been completed. Our ongoing evaluation will use a longitudinal, mixed-methods design to assess effectiveness, implementation outcomes, cost-effectiveness, and scalability.

Participants frequently have described mentorship and peer support as central to belonging and sustaining hope. Consistent with evidence that psychological needs shift across stages of recovery (152, 153), participants also emphasized the role of mentorship, career coaching, and GROW in later-stage recovery for supporting self-distinctiveness as work-related identities become more salient.

Although overall retention has been high, several participants reduced engagement after securing employment. This pattern may reflect limited structure or incentives for continued participation once immediate job-search goals were met. It may also reflect a group-process tension, insofar as a career focus can foreground differences in educational and vocational backgrounds rather than a shared recovery identity, potentially weakening the sense of belonging typically fostered by peer support.

Virtual delivery has reduced common barriers to participation and supported engagement. However, some participants have perceived the absence of a dedicated physical space—outside of Recovery Celebration Events—as limiting opportunities for informal social connection.

Finally, sustaining services under unstable funding constrained the strength and continuity of cross-organizational partnerships. Ongoing system-coordination demands—including role clarity, delineation of responsibilities, and communication protocols—created implementation challenges and underscored the need for collaborative infrastructure to support program continuity.

## Data Availability

The original contributions presented in the study are included in the article/supplementary material. Further inquiries can be directed to the corresponding author.

## Footnotes

* The RCs-36 (a 36-item instrument) has been developed jointly by R1 Learning and Dr. David Best and can be located at: https://r1learning.com/rcs36.
